# Complete Chloroplast Genomes of Five *Ulmus pumila* Cultivars: Comparative Analysis, Evolutionary Dynamics, and Germplasm Authentication Implications

**DOI:** 10.1002/ece3.74352

**Published:** 2026-09-29

**Authors:** Ning Wang, Aonan Yu, Xuan Ye, Leyi Zhang, Shuxiang Feng, Zhongfei Jiang, Chang Liu, Yongtan Li, Yinran Huang

**Affiliations:** ^1^ College of Forestry, College of Landscape and Tourism, Key Laboratory of National Forestry and Grassland Administration on Colorful Tree, Hebei Key Laboratory of Floral Biological Breeding Hebei Agricultural University Baoding China; ^2^ Hebei Forestry and Grassland Science Research Institute Shijiazhuang China

**Keywords:** chloroplast genome, comparative genomics, phylogenetic relationship, *Ulmus*

## Abstract

The complete chloroplast genomes of five *Ulmus pumila* cultivars (“Yangguang Nanhai,” “Yangguang Nühai,” “Xu Ri,” “Yang Gang,” and “Zhao Yang”) were sequenced and analyzed in this study, with publicly available *Ulmus pumila* accessions from NCBI included for intraspecific comparative analysis to elucidate cultivar‐level genomic variations, genetic diversity and phylogenetic placement. The chloroplast genomes showed typical circular quadripartite structures, ranging from 159,391 to 159,769 bp with GC contents of 35.47%–35.56%. Each genome encoded 131–133 genes, including 86–89 protein‐coding genes, 36–37 tRNAs, and 8 rRNAs, with minor intraspecific variations in gene content mainly attributed to tRNA pseudogenization. In total, 154–164 simple sequence repeats (SSRs) and 63–155 long repeats were identified, with SSRs predominantly comprising mononucleotide A/T repeats; cultivar‐specific repeat loci were detected that can serve as candidate markers for cultivar authentication. Nucleotide diversity analysis revealed hypervariable regions (e.g., *trnH‐GUG_psbA*, *ndhF_rpl32*) at the intraspecific level that could serve as complementary molecular markers for *Ulmus pumila* germplasm identification. IR boundary analysis revealed minor length fluctuations of boundary genes (e.g., *rps19*, *ycf1*) among cultivars, representing intraspecific structural polymorphism rather than major evolutionary events. In phylogenetic analysis, the five cultivars were clustered into a single well‐supported clade together with other *Ulmus pumila* accessions, providing robust support for the monophyly of the genus *Ulmus* and confirming the close maternal genetic background of these cultivated materials. Overall, this study presents the first complete chloroplast genome resource for these five *Ulmus pumila* cultivars, fills the gap of intraspecific plastome variation for cultivated *Ulmus pumila* germplasm, and establishes a molecular foundation for germplasm identification, maternal lineage tracing and genetic breeding of *Ulmus* species.

## Graphical abstract

We sequenced and characterized the complete chloroplast genomes of five *Ulmus pumila* cultivars, which possess typical circular quadripartite structures (159,391–159,769 bp, 35.47%–35.56% GC) encoding 131–133 genes and identified abundant SSRs, long repeats, and core hypervariable molecular markers (trnH‐GUG_psbA, ndhF_rpl32), while IR boundary and phylogenetic analyses revealed chloroplast genome evolutionary dynamics and the distinct phylogenetic position of these cultivars. As the first report of complete chloroplast genomes for these five *U. pumila* cultivars, this study significantly expands the Ulmus genomic resource pool and provides critical molecular support for their accurate germplasm identification, genetic improvement, and high‐value industrial application.

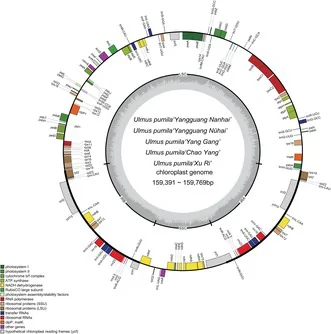

**Abbreviations**
BI: Bayesian Inference
BLASR: Basic Local Alignment with Successive Refinement
BLAST: Basic Local Alignment Search Tool
bp: base pair
EMBOSS: European Molecular Biology Open Software Suite
ENC: Effective Number of Codons
GC3s: GC Content at the Third Codon Position
IR: Inverted repeats
IRa: Inverted repeat a
IRb: Inverted repeat b
JLA: Junction between LSC and IRa
JLB: Junction between LSC and IRb
JSA: Junction between SSC and IRa
JSB: Junction between SSC and IRb
Ka: Non‐synonymous substitution rate
Ks: Synonymous substitution rate
LSC: Large single‐copy
MAFFT: Multiple Alignment using Fast Fourier Transform
MISA: MIcroSAtellite identification tool
ML: Maximum likelihood
mRNA: messenger Ribonucleic acid
OGDRAW: Organellar Genome DRAW
Pi: Nucleotide diversity
rRNA: ribosomal Ribonucleic acid
RSCU: Relative Synonymous Codon Usage
SSC: Small single‐copy
SSR: Simple sequence repeats
trimAL: trim Alignment
tRNA: transfer Ribonucleic acid
UTR: Untranslated Region

## 1 Introduction

The Ulmaceae sensu lato s.l. was first described by Mirbel in 1815, comprising two subfamilies: Ulmoideae and Celtidoideae (Zavada and Kim 1996; Wiegrefe et al. 1998). *Ulmus* species are mainly distributed in the Northern Hemisphere. Elm trees are excellent for environmental improvement due to their tolerance to cold, drought, and salt‐alkali stress (Caudullo and De Rigo 2016; Zuo et al. 2017). Their bark, leaves, and fruits have long been used as sedative and diuretic medicines, holding immense potential as industrial raw materials (Gu et al. 2019). Most *Ulmus* woods are hard, delicate, wear‐resistant, and tough, making them high‐quality materials for furniture, construction, and bridges. The bark and root bark of elms contain beneficial substances such as flavonoids, many of which possess significant medicinal value (Cheng et al. 2020; Jung et al. 2008). The phloem of elms has high viscosity and can be used as a natural plant adhesive, while leaves serve as animal feed (Beigh et al. 2021). Understanding their cp genomic features will facilitate targeted breeding for high‐quality wood and medicinal compounds.

Structural variations and abundant phylogenetic information in cp genomes are valuable for resolving the evolutionary relationships of complex species (Wicke et al. 2011; Moore et al. 2007). The cp genomes of most higher plants exhibit a typical quadripartite structure, consisting of a large single‐copy (LSC) region, a small single‐copy (SSC) region, and a pair of inverted repeats (IRs), which is highly conserved among species (Li et al. 2013). Due to their typical uniparental inheritance, moderate evolutionary rate, and ease of obtaining high‐quality sequences, cp genomes have been recognized as reliable molecular markers for studying plant phylogeny, species delimitation, and evolutionary history (Chen, Chen, et al. 2022; Chen, Hu, and Zhang 2022). Previous studies have analyzed the chloroplast structure, phylogeny and universal barcodes at the genus level of *Ulmus*. However, there is still a gap in the research on intraspecific chloroplast variations and species‐specific markers of cultivated varieties of *Ulmus pumila* (Liu, Cui, et al. 2025; Liu, Li, et al. 2022).

The five *Ulmus pumila* cultivars all originated from the selection of seedlings of *Ulmus pumila* . The selection was conducted in Shijiazhuang Hebei Forestry and Grassland Science Research Institute. They share common traits such as straight tree trunks and smooth bark of young trees. There are distinguishable differences in morphological characteristics such as tree crown shape, branch extension direction, color of 1‐year‐old branches, leaf size and texture, and tree bark color of young trees. The morphologies of the cultivated varieties of *Ulmus pumila* are highly similar, making traditional morphological identification prone to confusion. The resolution of the universal DNA barcodes is insufficient, and there is an urgent need for species‐specific markers based on the chloroplast genome and germplasm traceability tools. In this study, we sequenced, assembled, and annotated the cp genomes of *Ulmus pumila* “Yangguang Nanhai,” “Yangguang Nühai,” “Xu Ri,” “Yang Gang,” and “Chao Yang,” and compared them with closely related species. Phylogenetic trees were constructed using cp genomes to deepen our understanding of the evolution of Ulmaceae species. The uniparental inheritance, moderate mutation rate, and relative ease of sequencing make cp genomes more effective than nuclear and mitochondrial genomes for exploring plant origin and evolution, clarifying phylogenetic relationships among different taxa, and species identification (Chen, Chen, et al. 2022; Chen, Hu, and Zhang 2022; Ye et al. 2024).

## 2 Materials and Methods

### 2.1 Plant Material and Sequencing

Five *Ulmus* cultivars, namely *Ulmus pumila* “Yangguang Nanhai,” “Yangguang Nühai,” “Xu Ri,” “Yang Gang,” and “Chao Yang”, were collected from the Hebei Academy of Forestry and Grassland Sciences in Shijiazhuang, China (114°28′12″ E, 38°08′23″ N). In May 2025, leaves were preserved in liquid nitrogen. Both short‐read (Illumina) and long‐read sequencing (PacBio Sequel II) technologies were used in this study. Shanghai Lingen Biotechnology Co. Ltd. was commissioned to construct and sequence long‐fragment libraries and short‐fragment (~500 bp) libraries of cp DNA using the PacBio Sequel II and Illumina NovaSeq 6000 platforms, respectively. Raw data were quality‐controlled with FastQC and filtered with Trimmomatic to ensure the reliability of subsequent assembly and analysis.

### 2.2 Genome Assembly and Annotation

A dual‐strategy approach was adopted for cp genome assembly based on Illumina and PacBio sequencing data. Short‐read data were initially assembled using GetOrganelle (Jin et al. 2020). Potential cp contigs were selected via BLAST alignment, and PacBio long reads were mapped to the reference using BLASR (Camacho et al. 2009). Gene annotation was ultimately completed using the online tool GeSeq, and gene structures were verified by BLAST and manual correction. Genome maps were generated using OGDRAW (Tillich et al. 2017; Lohse et al. 2007). The Relative Synonymous Codon Usage (RSCU) was calculated using the cusp program (EMBOSS v6.6.0.0) to assess codon preference, and heatmaps were plotted with TBtools 1.116 (Sharp and Li 1986; Chen et al. 2020). The Effective Number of Codons (ENC) of the cp genome was analyzed using CodonW software to evaluate codon usage bias (Ling et al. 2024).

### 2.3 Long Repeat and SSR Analysis

To identify long repeat sequences in the cp genome, REPuter software was used to detect four types: forward, reverse, palindromic, and complement repeats (Kurtz et al. 2001). Parameters were set as follows: minimum repeat length of 30 bp, maximum of 1000 bp, Hamming distance of 3, and sequence identity > 90%. Simple sequence repeat (SSR) analysis was performed using MISA with the following criteria: mononucleotide repeats ≥ 8, dinucleotide and trinucleotide repeats ≥ 5, and tetra‐to‐hexanucleotide repeats ≥ 3. Primers for identified SSR loci were designed using Primer3 (Guo et al. 2017).

### 2.4 Comparative Chloroplast Genomic Analysis and Contraction/Expansion of IR Regions

Seventeen additional cp genome sequences of other Ulmus members were downloaded from the NCBI database for comparative analysis, with a focus on intraspecific variation among *Ulmus pumila* germplasms. A total of 22 cp genomes were analyzed using mVISTA software (Hejazi et al. 2025). Nucleotide variability (Pi) of coding genes and non‐coding regions was analyzed with DnaSP v5.10 to identify divergent hotspot regions (Librado and Rozas 2009). The expansion and contraction of IR/SC junctions across cp genomes were compared using IRscope (Amiryousefi et al. 2018).

### 2.5 Phylogenetic Analysis

Phylogenetic analysis was conducted based on cp genome sequences of 28 species (including 25 Ulmaceae species). Multiple sequence alignment was performed with MAFFT, followed by trimming with trimAL (Katoh et al. 2002). Maximum likelihood (ML) phylogenetic trees were constructed using PhyML v3.0 with 1000 bootstrap replicates (Guindon et al. 2010). Ka/Ks ratios were determined using KaKs_Calculator (Zhang et al. 2020). To identify conserved blocks and structural variations among cp genomes, genome‐wide synteny comparison was performed using Mauve software to detect potential rearrangement events (Wang et al. 2025).

## 3 Results

### 3.1 Chloroplast Genome Structure and Gene Content

The total cp genome lengths of the five *Ulmus* cultivars ranged from 159,391 bp ( *Ulmus pumila* “Yangguang Nühai”) to 159,769 bp ( *Ulmus pumila* “Yangguang Nanhai”), with GC contents of 35.47%–35.56% (Figure 1). All five cp genomes exhibited a typical circular quadripartite structure, consisting of a large single‐copy (LSC) region (87,972–88,352 bp), a small single‐copy (SSC) region (18,759–18,841 bp), and a pair of inverted repeats (IRa and IRb, 26,288–26,330 bp). A total of 131–133 genes were annotated, including 86–89 protein‐coding genes, 36–37 tRNA genes, and 8 rRNA genes (Table 1). Specifically, “Yangguang Nanhai” contained 131 genes, “Yang Gang” contained 132 genes, and “Yangguang Nühai,” “Xu Ri” and “Zhao Yang” each contained 133 genes. The intraspecific variation in gene count was mainly caused by the pseudogenization or partial deletion of one tRNA gene in the LSC region, which represents a common intraspecific structural polymorphism in plant chloroplast genomes. A total of 16 genes contained introns, including 14 with one intron (e.g., *rps16*, *atpF*) and 2 with two introns (*clpP*, *ycf3*), consistent with other *Ulmus* species (Liu, Cui, et al. 2025; Liu, Li, et al. 2022).

**FIGURE 1:**
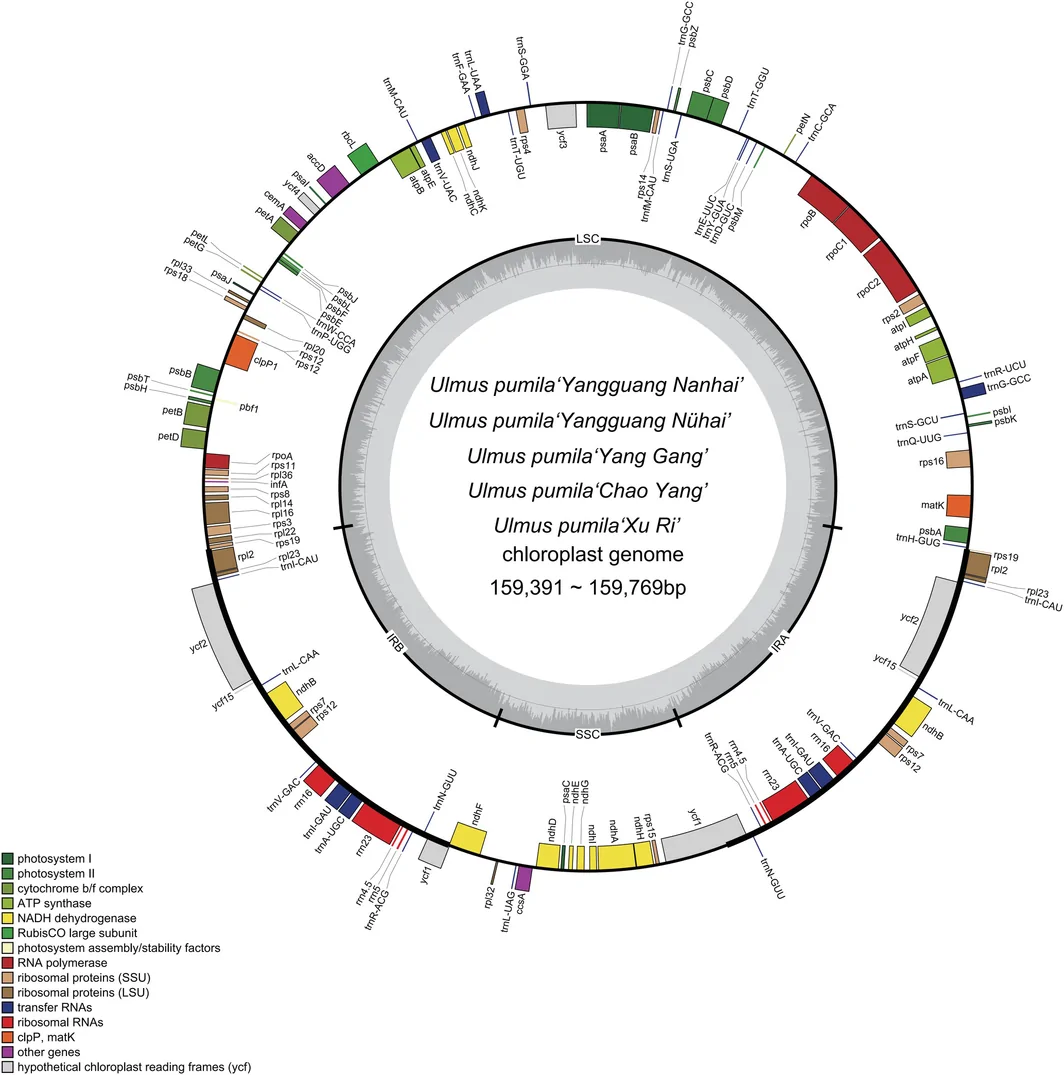
Chloroplast genome map of *Ulmus* spp. The dark gray in inner circle corresponds to GC content, and light gray represents AT content.

**TABLE 1:** Basic information of the chloroplast genomes of *Ulmus* spp.

Species	YGNH‐B	YGNH‐G	XR	YG	ZY
Length (bp)	159,769	159,391	159,398	159,756	159,656
Total gene number	131	133	133	132	133
GC content (%)	35.48%	35.56%	35.56%	35.47%	35.49%
LSC length (bp)	88,352	87,972	87,979	88,358	88,258
SSC length (bp)	18,841	18,759	18,759	18,822	18,802
IRa length (bp)	26,288	26,330	26,330	26,288	26,298
IRb length (bp)	26,288	26,330	26,330	26,288	26,298
mRNA genes	86	89	89	88	89
rRNA genes	8	8	8	8	8
tRNA genes	37	36	36	36	36
Intergenic region length (bp)	79,279	78,886	78,893	85,005	79,235
mRNA total length (bp)	80,490	80,505	80,505	74,751	80,421

*Note:* YGNH‐B, *Ulmus pumila* “Yangguang Nanhai”; YGNH‐G, *Ulmus pumila* “Yangguang Nühai”; XR, *Ulmus pumila* “Xu Ri”; YG, *Ulmus pumila* “Yang Gang”; ZY, *Ulmus pumila* “Zhao Yang”.

All five *Ulmus* cultivars included in this study were selected and bred from seedling progenies. To explore the potential correlation between chloroplast genomic variations and phenotypic differentiation among cultivars, the key morphological diagnostic traits of the five cultivars are summarized in Table 2. When compared with other *Ulmus* accessions deposited in the NCBI GenBank, the five cultivars in this study retained the conserved quadripartite chloroplast genome architecture, but exhibited obvious intraspecific structural variations in genome size, IR boundary shifts, and gene content. Variation in gene number among cultivars, caused by tRNA gene pseudogenization or partial deletion in the LSC region, is an important manifestation of intraspecific chloroplast genome diversity in *Ulmus* cultivars, and these genomic variations may be associated with the distinct morphological traits of different cultivars.

**TABLE 2:** Morphological diagnostic characteristics of five *Ulmus pumila* cultivars.

Species	Trunk and crown	Leaf	Samara and seed
YGNH‐B	Straight trunk form, smooth bark surface	Typical ovate leaf shape, standard green color	No obvious difference
YGNH‐G	Relatively poor trunk straightness, spreading crown shape	Typical ovate leaf shape, standard green color	Abundant samara production
XR	Reddish trunk appearance, upright growth habit	Enlarged and thickened leaf blades, dark green color	No obvious difference
YG	Narrow fastigiate crown, upright and fascicled branches	Typical ovate leaf shape, standard green color	No obvious difference
ZY	Medium trunk straightness, open crown	Lanceolate willow‐shaped leaves, yellow‐green leaf color	No obvious difference

*Note:* YGNH‐B, *Ulmus pumila* “Yangguang Nanhai”; YGNH‐G, *Ulmus pumila* “Yangguang Nühai”; XR, *Ulmusum pila* “Xu Ri”; YG, *Ulmus pumila* “Yang Gang”; ZY, *Ulmus pumila* “Zhao Yang”.

### 3.2 SSRs and Long Repeat Sequences

A total of 154–164 SSRs were identified in six *Ulmus pumila* cultivars (five newly sequenced cultivars plus the reference cultivar “Jinmi” retrieved from NCBI), with 159 SSRs detected in “Zhao Yang” (Table S1). SSR types included mononucleotide to hexanucleotide repeats, with mononucleotide repeats being absolutely dominant. Among dinucleotide repeats, AT/AT was the most common type. For pentanucleotide repeats, only TTATT was present in *Ulmus pumila* “Yangguang Nühai” and “Xu Ri,” while TAAAG was detected in all six cultivars. The hexanucleotide repeat TGGTAG was only found in “Yangguang Nühai” and “Xu Ri.” Overall, the number of SSRs decreased with increasing repeat unit length, and A/T bases dominated the repeat units, showing a distinct base preference (Figure 2A).

**FIGURE 2:**
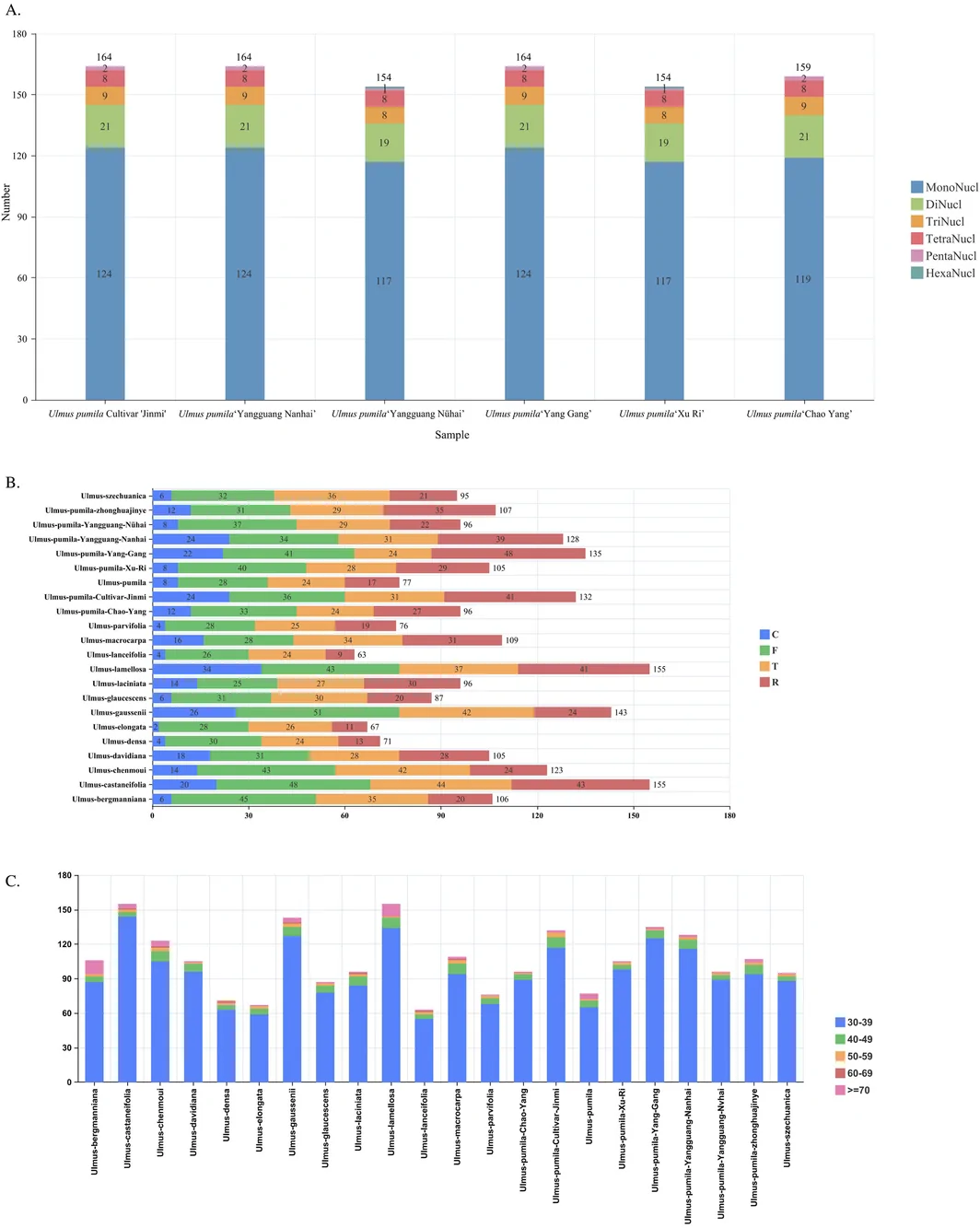
Type and number of SSRs (A) and long repeat sequences (B and C) in the CP genomes of *Ulmus* spp.

For long repeat sequences with length ≥ 30 bp in 22 cp genomes, 63 (*Ulmus* lanceifolia) to 155 (*Ulmus* castaneifolia, *Ulmus* lamellosa) fragments were identified (Figure 2B). Within *Ulmus pumila* , the number of long repeats ranged from 96 to 135 among cultivars, with forward and palindromic repeats being the dominant types. Repeat sequences mainly included F (Forward), R (Reverse), C (Complement), and P (Palindromic). The number of C repeats was the lowest in *Ulmus elongata* (2) and the highest in *Ulmus lamellosa* (34). Most of these repeat fragments ranged in length from 30 to 39 bp, while sequences of 60–69 bp were rare. *Ulmus bergmanniana* (12) and *Ulmus lamellosa* (11) had the highest number of repeat sequences with length ≥ 70 bp (Figure 2C).

### 3.3 Comparative Genomic and Nucleotide Polymorphism

Global alignment of *Ulmus* cp genomes using mVISTA was performed to analyze the distribution of conserved and variable sites. The results showed that the overall sequence structure of *Ulmus* cp genomes was relatively conserved (Figure 3). In the selected *Ulmus* samples, *Ulmus pumila* “Yangguang Nanhai”, “Yangguang Nühai”, “Xu Ri”, “Yang Gang”, “Chao Yang”, and the cultivated variety *Ulmus pumila* Cultivar “Jinmi” exhibited high sequence similarity, as did *Ulmus parvifolia* and *Ulmus laciniata*. Certain species, such as *Ulmus glaucescens*, *Ulmus szechuanica*, *Ulmus densa*, *Ulmus gaussenii*, *Ulmus chenmoui*, *Ulmus lanceifolia*, *Ulmus bergmanniana*, and *Ulmus macrocarpa*, showed some sequence differences. Variable sites were mainly distributed in the LSC (Large Single‐Copy) and SSC (Small Single‐Copy) regions, with non‐coding regions showing significantly higher variability than coding regions. The four rRNA genes (rrn23, rrn16, rrn5, and rrn4.5) all exhibited high conservation in the global alignment.

**FIGURE 3:**
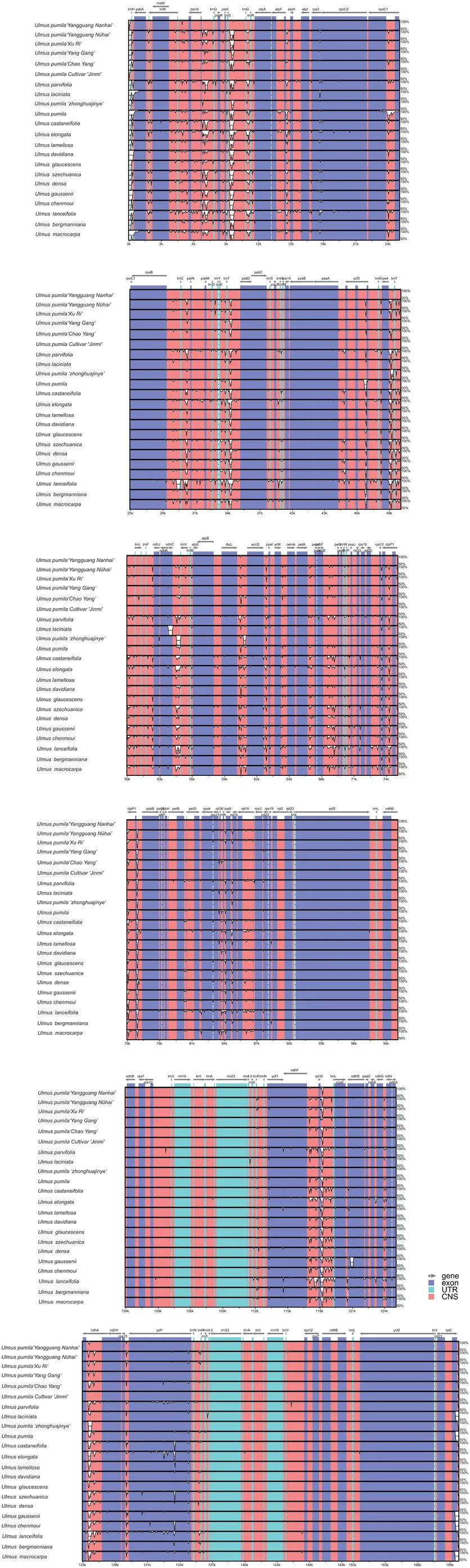
Global alignment of the CP genomes of *Ulmus* spp.

To explore the variable regions in the cp genome sequences of *Ulmus* species, nucleotide diversity (Pi) analysis was performed. At the intraspecific level of *Ulmus pumila* , overall Pi values were lower than 0.01, reflecting high sequence conservation among cultivars; nevertheless, several hypervariable intergenic regions including *trnH‐GUG_psbA* and *ndhF_rpl32* showed higher polymorphism than universal DNA barcodes and can be used for cultivar differentiation. At the interspecific level, the Pi values of the entire cp genome ranged from 0 to 0.05, with more nucleotide diversity peaks in the LSC and SSC regions, while the IR regions were relatively conserved. Higher Pi values were observed in several genes or intergenic regions, including *trnH‐GUG_psbA*, *trnS‐GCU_trnG‐UCC*, the exon spacing region of *rpoC1*, *trnT‐GGU_psbD*, the exon region of *ycf3, ndhC_trnV‐UAC*, and adjacent regions such as *ndhF_rpl32*, *rpl32_trnL‐UAG*, and *ndhD* (Figure 4). These regions with high sequence variation, especially those located in non‐coding regions or intergenic regions, may serve as potential molecular markers for *Ulmus* species identification and phylogenetic studies.

**FIGURE 4:**
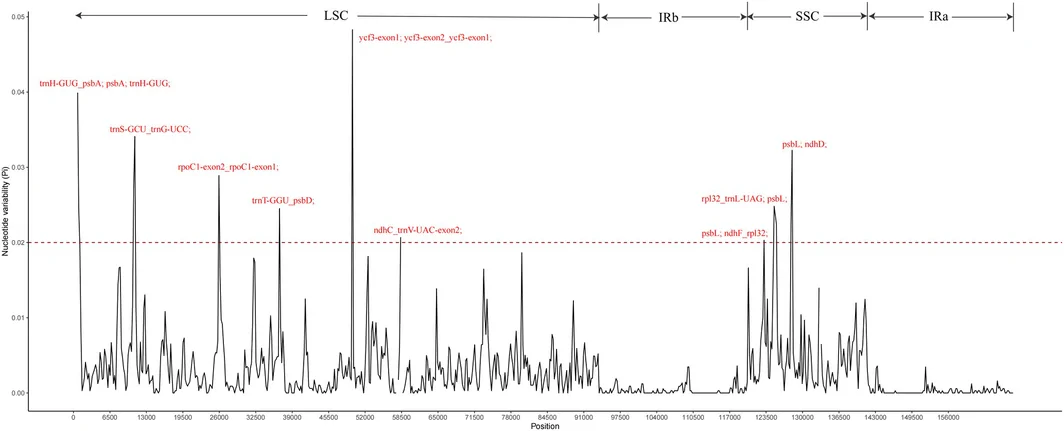
Nucleotide diversity (Pi) across the chloroplast genomes of 22 Ulmus species. The x‐axis indicates the genome position, with four structural regions (LSC, IRb, SSC, IRa) labeled above. The y‐axis represents nucleotide diversity values. Analysis was performed with a 300 bp window and 200 bp step size. Peaks correspond to hypervariable intergenic and genic regions.

The cp genomes of Ulmaceae plants have a typical quadripartite structure, with total lengths ranging from 157,356 bp (*Trema levigatum*) to 160,388 bp (*Ulmus lamellosa*) (Figure 5). The length of the inverted repeat region is relatively conserved among species, with IRa and IRb mostly ranging from 26,000 to 26,500 bp. Some species, such as *Ulmus lamellosa*, show slight asymmetry between IRb (26,507 bp) and IRa (26,402 bp). Within *Ulmus pumila* cultivars, IR boundary genes showed only minor length fluctuations (within 50 bp), and no large‐scale IR expansion or contraction was detected. These small boundary shifts represent intraspecific genetic polymorphism rather than major evolutionary events. At the IR‐LSC boundary (JLB), the *rps19* gene is located near the boundary in most species, with lengths ranging from 184 bp (*Ulmus castaneifolia*) to 279 bp (multiple species) in the IRb region. The *rpl2* gene crosses the JLB into the IRb region in multiple *Ulmus* species, with a length of 1510 bp. At the IR‐SSC boundary (JSA), the *ycf1* gene generally crosses the JSA, with lengths ranging from 3687 bp (*Ulmus castaneifolia*) to 4671 bp (*Pteroceltis tatarinowii*) in the IR region and 1091 bp (*Trema levigatum*) to 1576 bp (*Ulmus chenmoui*) in the SSC region. The *ndhF* gene is located near the JSB, with lengths ranging from 2155 bp (*Ulmus lanceifolia*) to 2252 bp ( *Celtis sinensis* ) in the SSC region. The *trnH* gene is located on the LSC side of the JLA boundary, with distances from the boundary ranging from 0 bp ( *Celtis sinensis* ) to 535 bp (*Ulmus gaussenii*). The *psbA* gene appears in the JLA region of some species, such as *Ulmus davidiana* and *Ulmus gaussenii*, with a length of 1062 bp. The *rpl23* gene is located near the JLA in species such as *Ulmus laciniata* and *Ulmus macrocarpa*, with a length of 282 bp.

**FIGURE 5:**
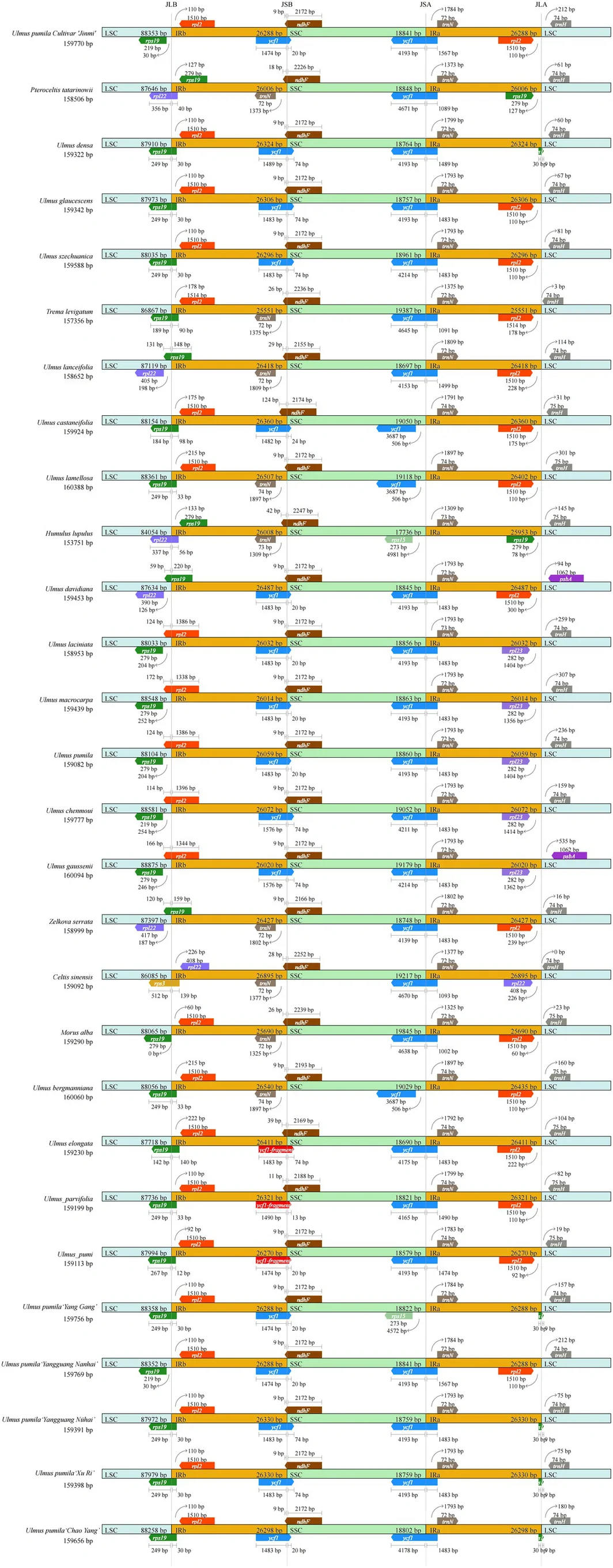
Comparison of the LSC, IR, and SSC border regions among 28 Ulmaceae species chloroplast genomes. (JLB, JSB, JSA, and JLA are the junction boundaries between LSC/IRb, SSC/IRb, SSC/IRa, and LSC/IRa, respectively.)

Interspecific differences in the distribution and length of genes at the IR boundaries reflect local expansion, contraction, and structural rearrangement events in the cp genomes of Ulmaceae plants during evolution, while intraspecific boundary variations among *Ulmus pumila* cultivars have limited evolutionary significance but can serve as auxiliary markers for cultivar identification.

### 3.4 Codon Usage Bias Analysis

Analysis of amino acid coding frequencies in the cp genomes of 22 *Ulmus* taxa showed that the coding patterns of all taxa were highly consistent with no significant differences. In terms of amino acid coding frequency, the codon encoding leucine (Leu) had the highest usage frequency among the 22 *Ulmus* taxa, ranging from 0.1042 ( *Ulmus davidiana* ) to 0.1068 ( *Ulmus pumila* “Yang Gang”), while the codon encoding cysteine (Cys) had the lowest frequency, ranging from 0.0111 ( *Ulmus davidiana* ) to 0.0117 ( *Ulmus parvifolia* ). Methionine (Met) and tryptophan (Trp) had no synonymous codons to choose from, and their coding frequencies were stable (Figure 6A). The coding frequencies of other amino acids fluctuated minimally, further reflecting the conservation of the coding characteristics of this genome in *Ulmus*.

**FIGURE 6:**
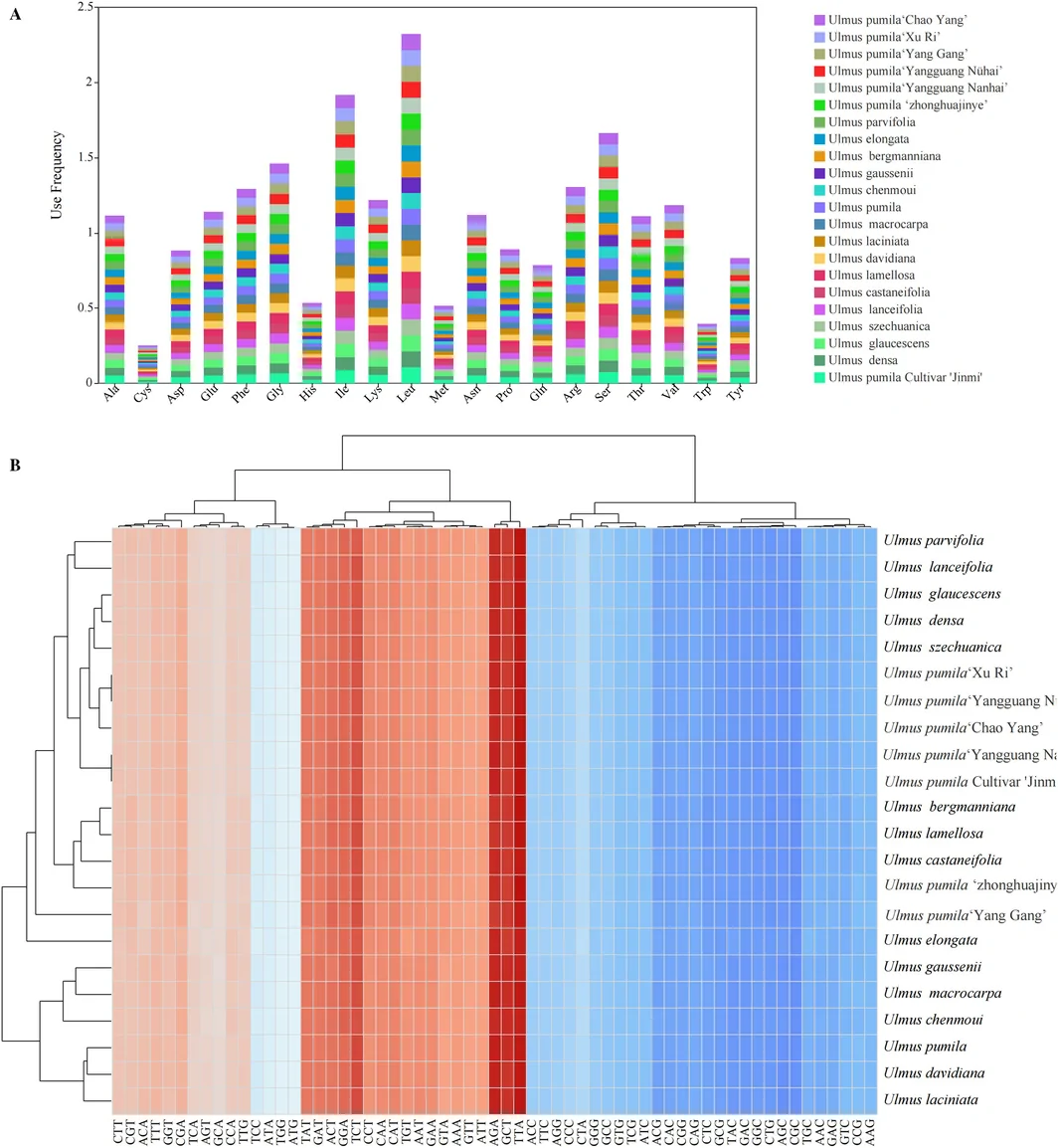
Frequency of amino acids (A) encoded in all protein‐coding genes, and the heat map (B) of RSCU values.

The Relative Synonymous Codon Usage (RSCU) patterns of synonymous codons in the cp genomes of 22 *Ulmus* taxa were highly consistent, with no significant interspecific differences and strong conservation (Figure 6B). Preferential codons with RSCU > 1.00 were mostly ending with A/U, while codons with RSCU < 1.00 were mostly ending with G/C, reflecting the preference for A/U‐ending codons in the cp genome.

Analysis of the ENC in the cp genomes of 22 *Ulmus* taxa showed that ENC values were distributed within the standard range of 20–60, and the ENC values of various *Ulmus* taxa were concentrated in the medium‐high range, indicating that the overall codon usage bias of their cp genomes was weak (Figure 7). The consistency of ENC values further confirms the conservation of the coding characteristics of *Ulmus* chloroplast genomes.

**FIGURE 7:**
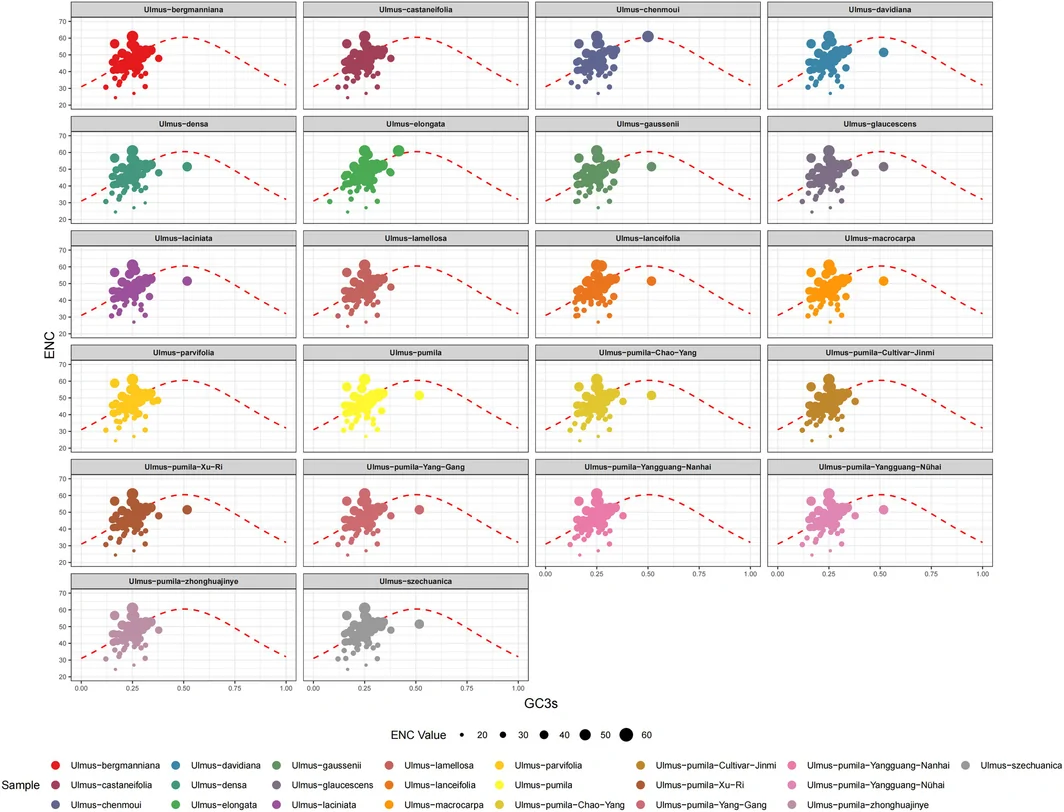
Scatter plot of Effective Number of Codons (ENC) vs. GC Content at the Third Codon Position (GC3s) for chloroplast genomes of *Ulmus* species.

### 3.5 Phylogenetic and Collinearity Analysis

To clarify the phylogenetic relationships among various taxa within the Ulmaceae family, this study constructed phylogenetic trees using the maximum likelihood (ML) and Bayesian inference (BI) methods based on the complete cp genome data of 28 Ulmaceae plants, with 6 closely related genera such as *Morus* and *Celtis* selected as outgroups. The topological structures obtained by the two analysis methods were highly consistent, significantly enhancing the credibility of phylogenetic inferences.

Phylogenetic analysis results showed that all *Ulmus* plants could be clearly divided into three main clades, reflecting a stable phylogenetic structure within the genus (Figure 8). Among them, *Ulmus elongata* was located at the basal position in the phylogenetic tree, suggesting that it may be an early diverging taxon in *Ulmus*. *Ulmus parvifolia* , *Ulmus lanceifolia*, and *Ulmus chenmoui* clustered into one clade, showing a close genetic relationship. Another main clade included *Ulmus davidiana* , *Ulmus laciniata*, *Ulmus macrocarpa*, and other species with relatively similar leaf morphological characteristics. All five *Ulmus pumila* cultivars clustered tightly within the *Ulmus pumila* clade and were clearly separated from other *Ulmus* species. This result indicates that the five cultivars share a highly consistent maternal genetic background derived from *Ulmus pumila* , with only subtle differentiation from wild *Ulmus pumila* accessions.

**FIGURE 8:**
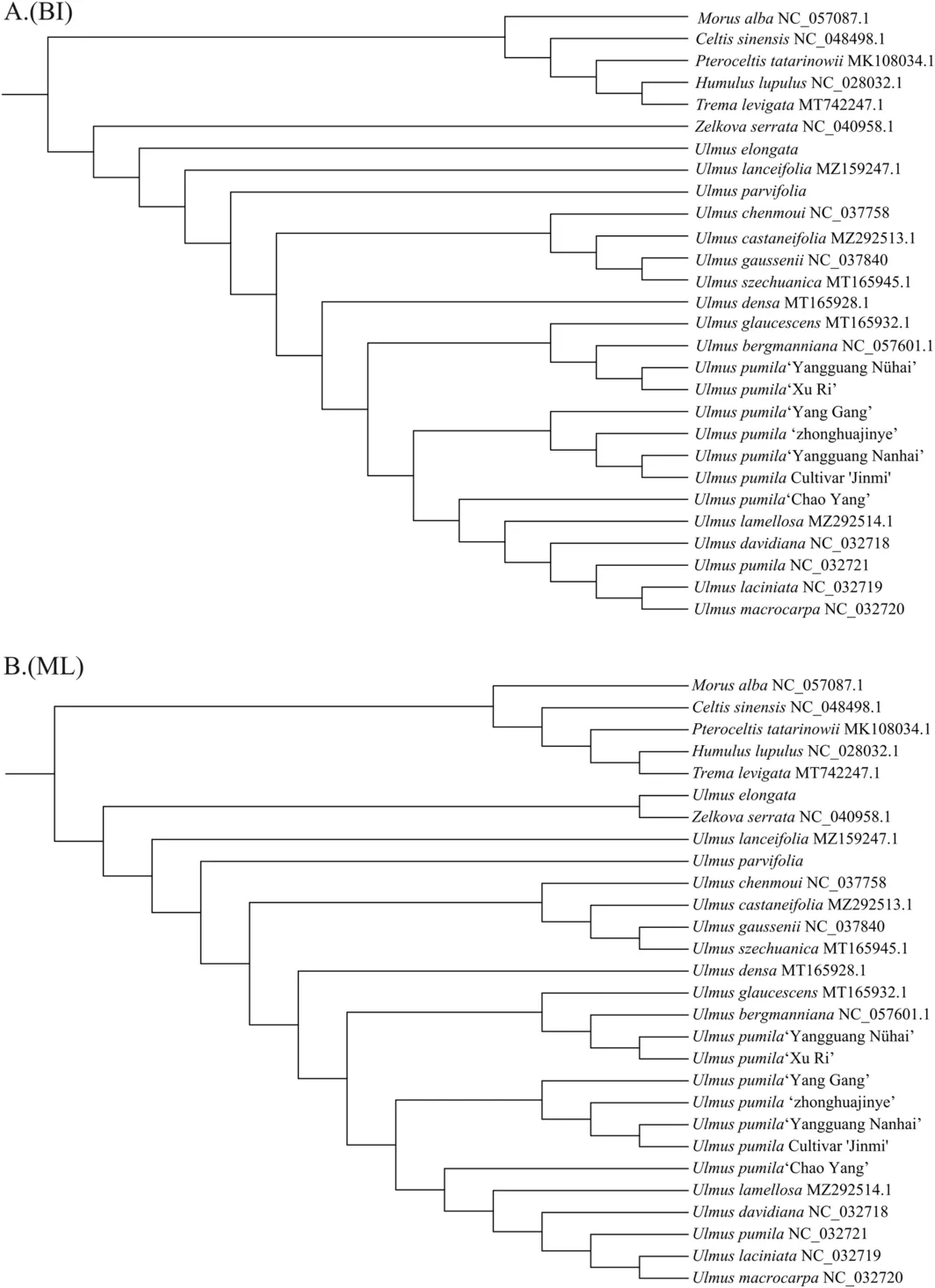
Phylogenetic trees of 28 Ulmaceae species. (A) BL法; (B) ML法.

Analysis of Ka (non‐synonymous substitution rate) and Ks (synonymous substitution rate) of shared protein‐coding genes in the cp genomes of 22 *Ulmus* plants showed that the Ka and Ks values of all genes were at a low substitution level of 0–0.05, and the Ks values of each gene were overall higher than the Ka values, resulting in Ka/Ks ratios all less than 1 (Figure 9). The uniformly low Ka/Ks ratios indicate that all shared protein‐coding genes are under strong purifying selection, with no signal of rapid adaptive evolution, which is a typical feature of angiosperm chloroplast genomes. The low substitution level further reflects the high conservation of the genetic characteristics of *Ulmus* chloroplast genomes.

**FIGURE 9:**
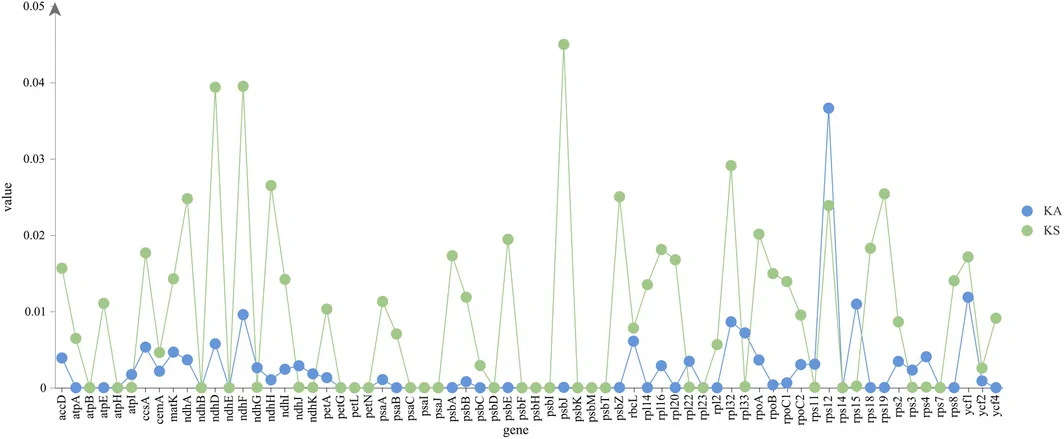
Selection pressure analysis of shared protein‐coding genes in *Ulmus* chloroplast genomes. Ka (non‐synonymous substitution rate) and Ks (synonymous substitution rate) values are shown on the y‐axis, with gene names on the x‐axis. All genes show Ka/Ks < 1 and extremely low substitution rates, indicating strong purifying selection and high sequence conservation.

Collinearity analysis of *Ulmus* chloroplast genomes showed that there were continuous and dense homologous gene connections in most regions among taxa, and the collinearity of core functional gene clusters such as photosynthesis and energy metabolism was highly conserved, reflecting the stability of genome structure (Figure 10). Only a few gene arrangement differences occurred in local regions, which may be caused by gene inversion or microdeletion (Table 1).

**FIGURE 10:**
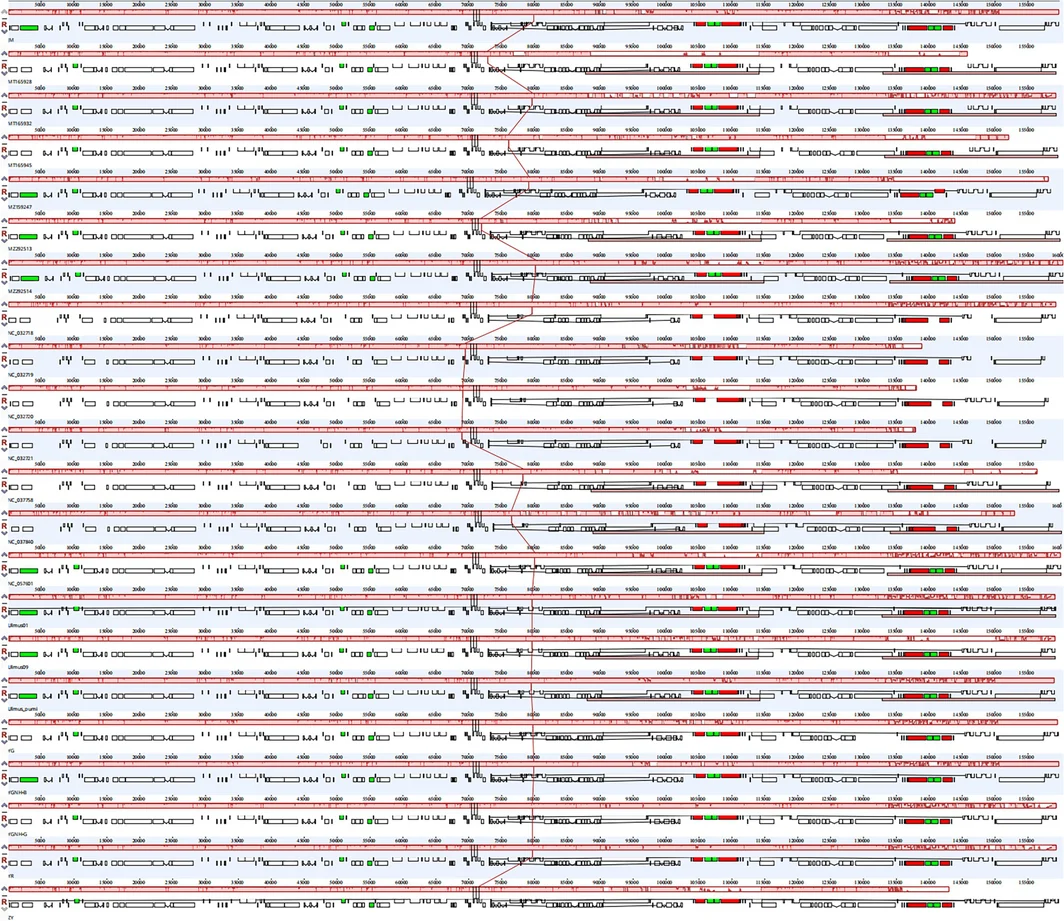
Analysis diagram of the linear alignment of the chloroplast genome.

## 4 Discussion

As a widely cultivated tree species with important ecological and economic value, *Ulmus pumila* has abundant cultivar resources, but its intraspecific plastome variation has rarely been systematically characterized. The typical quadripartite structure of its chloroplast genome, including two IR regions, one LSC and one SSC region, is consistent with the conservative characteristics of higher plant chloroplast genomes (Zhang et al. 2018) and matches the structural features reported for other *Ulmus* species (Liu, Cui, et al. 2025; Liu, Li, et al. 2022). The GC content is stable at 35.47%–35.56%, and the number of genes is 131–133. Core photosynthetic genes (e.g., psbA, atpF) and stress‐related genes (e.g., ndhA, rpl2) are highly conserved among cultivars, ensuring basic photosynthetic efficiency and growth stability. The complex agronomic traits such as wood properties, biomass yield and secondary metabolite content are mainly regulated by nuclear genes, and chloroplast genomes provide basic metabolic support.

Previous chloroplast genome studies of the genus *Ulmus* have mainly focused on interspecific phylogeny and structural variation at the genus level. This study extends the research scope to the cultivar level of *Ulmus pumila* . Five complete chloroplast genomes of *Ulmus pumila* cultivars are reported for the first time, substantially expanding the available genomic resources for cultivated elm germplasm. Intraspecific plastome variation among *Ulmus pumila* cultivars, including gene content differences, repeat sequence polymorphisms, and hypervariable regions, is characterized, providing finer‐scale variation information than previously documented interspecific differences. The utility of chloroplast markers for cultivar‐level authentication is assessed, delivering practical molecular tools for germplasm management and cultivar protection of *Ulmus pumila* .

Analysis of codon usage bias helps clarify gene function and evolutionary processes (Yang et al. 2024). Among the 22 *Ulmus* taxa in this study, leucine (Leu) is the most frequently encoded amino acid, while cysteine (Cys) has the lowest coding frequency. This pattern is common in similar plants but also shows intergeneric differences (Somaratne et al. 2020; Yang et al. 2014). Relative Synonymous Codon Usage (RSCU) analysis revealed a significant preference for A/U‐ending codons in the cp genomes of the five cultivars, with 31 preferential codons (RSCU > 1.00) mostly ending in A/U, which is a common feature of most land plant cp genomes. Meanwhile, the ENC values were concentrated in the medium‐high range, indicating an overall weak codon usage bias in the cp genomes of the five cultivars. This weak codon bias suggests that the cp genomes of these *Ulmus pumila* cultivars are subject to strong purifying selection during evolution, with random genetic drift having a weaker impact on codon usage patterns, which further supports the high conservation of the coding characteristics of *Ulmus* cp genomes.

A total of 154–164 SSRs were detected in this study. Due to their high polymorphism and codominance, SSRs are often used as effective genetic markers in population genetics and evolutionary studies (Li et al. 2023). In this study, mononucleotide A/T repeats are dominant in *Ulmus pumila* cultivars, and the number of long repeat sequences (≥ 30 bp) ranges from 63 to 155, with sequences of 30–39 bp being the most common, which is similar to findings in other plants (Wang et al. 2024; Zalapa et al. 2008). These repeat sequences, especially SSRs abundant in non‐coding regions, provide resources for the development of molecular markers for *Ulmus* species identification and genetic diversity analysis. For long repeat sequences with length ≥ 30 bp, forward and palindromic repeats were the main types, and most fragments were 30–39 bp in length. These repeat sequences are important drivers of cp genome rearrangement, sequence variation, and nucleotide polymorphism, and their interspecific and inter‐cultivar differences also provide valuable information for understanding the evolutionary dynamics of *Ulmus* cp genomes.

DNA barcoding technology plays an important role in species identification (Liu et al. 2019). Although fragment combinations such as matK+ rbcL are widely used (Algarni 2022), the resolution ability of a single fragment is limited, and the use of multi‐fragment combinations or highly variable single genes has received increasing attention (Xu et al. 2024). In this study, nucleotide diversity (Pi) analysis revealed that hypervariable regions including *trnH‐GUG_psbA* and *ndhF_rpl32* had significantly higher Pi values than universal barcodes, and these regions were mainly located in non‐coding intergenic regions of the LSC and SSC regions, which are more sensitive to environmental selection and have faster evolutionary rates (Dong et al. 2015). Notably, the ycf1 gene, with a high Pi value of 0.035, also showed significant length variation at the IR/SSC boundary among different *Ulmus* species and cultivars, which further supports its potential as a core barcode for *Ulmus* species identification at low taxonomic levels.

Chloroplast genomes are predominantly maternally inherited in most angiosperms, including *Ulmus*. Therefore, the hypervariable markers identified in this study can only reflect maternal lineage differentiation and are insufficient to detect nuclear hybridization or introgression events, which are common in elm breeding programs. These chloroplast markers are recommended as a complementary tool for germplasm purity testing and maternal origin tracing, and should be combined with nuclear markers (e.g., genomic SSRs, SNPs) for comprehensive identification of hybrid cultivars.

The IR region is the most conserved region in the chloroplast genome, crucial for maintaining genome structural stability and preserving essential genes (Gu et al. 2022). Comparison of IR/SC junctions showed that the distribution and length of boundary genes such as rps19, ycf1, and ndhF differed significantly among different Ulmaceae species, and the ycf1 gene crossed the JSA boundary in all five cultivars with varying lengths, which directly reflects the expansion and contraction events of the IR region during the evolution of *Ulmus* (Guo et al. 2021). Among *Ulmus pumila* cultivars, only minor boundary fluctuations were observed, which are more likely to be intraspecific genetic polymorphisms rather than independent evolutionary events. These subtle variations can still be used as auxiliary molecular features for cultivar differentiation. Phylogenetic analysis based on complete cp genomes using ML and BI methods showed highly consistent topological structures, with all five *Ulmus pumila* cultivars closely clustered into an independent and stable monophyletic clade, which is significantly differentiated from wild *Ulmus* species. This result indicates that the five cultivars have a highly consistent genetic background derived from *Ulmus pumila* , and have formed significant genetic differentiation from wild relatives during artificial selection and breeding. Meanwhile, the phylogenetic results strongly support that *Ulmus* is a monophyletic group, which is consistent with previous systematic studies of Ulmaceae based on cp genome data. Ka/Ks ratio analysis showed that the Ka/Ks values of all shared protein‐coding genes were less than 1, indicating that these genes are generally under strong purifying selection during evolution, which further confirms the functional conservation of *Ulmus* cp genomes from the perspective of evolutionary selection pressure, and also ensures the reliability of cp genomes as a tool for phylogenetic analysis of *Ulmus*.

The *Ulmus pumila* cultivars involved in this study, as stress‐tolerant tree species, have potential for popularization and application in arid and saline‐alkali ecological restoration and agroforestry composite systems (Zhang et al. 2025). The five cultivars analyzed in this study differ in morphological diagnostic traits including crown shape, leaf color, and growth rate, which are the main criteria for phenotypic identification. The cultivar‐specific chloroplast variations identified in this study, including specific SSR loci and hypervariable region haplotypes, provide molecular markers corresponding to these morphological features, enabling accurate cultivar identification independent of growth stage. The identified SSR markers can be used for cultivar authentication and genetic linkage map construction, facilitating the breeding process of *Ulmus pumila* . The chloroplast genomic resources obtained in this study lay a foundation for germplasm resource evaluation, maternal lineage analysis and molecular‐assisted breeding of *Ulmus pumila* , and also provide reference data for further evolutionary research of the genus *Ulmus*.

## 5 Conclusions

In this study, the complete chloroplast genomes of five *Ulmus pumila* cultivars were newly assembled and annotated, and intraspecific comparative analysis was performed with publicly available *Ulmus pumila* accessions from NCBI. These genomes exhibited a typical conserved quadripartite structure, with consistent characteristics in length, GC content, gene composition, and codon usage pattern across taxa. Hypervariable regions including trnH‐GUG_psbA and ndhF_rpl32 were identified as potential molecular markers for *Ulmus* species identification. Phylogenetic analysis supported *Ulmus* as a monophyletic group, with the five cultivars clustered into an independent clade. Our findings enrich *Ulmus* genomic resources and provide a solid molecular basis for its germplasm authentication and genetic breeding.

## Author Contributions

**Ning Wang:** formal analysis (equal), investigation (equal), methodology (equal), writing – original draft (equal), writing – review and editing (equal). **Aonan Yu:** data curation (equal), formal analysis (equal), investigation (equal). **Xuan Ye:** data curation (equal), investigation (equal). **Leyi Zhang:** data curation (equal), investigation (equal), methodology (equal). **Shuxiang Feng:** data curation (equal), investigation (equal). **Zhongfei Jiang:** data curation (equal), investigation (equal), methodology (equal). **Chang Liu:** data curation (equal), investigation (equal), methodology (equal). **Yongtan Li:** conceptualization (equal), project administration (equal), writing – review and editing (equal). **Yinran Huang:** conceptualization (equal), funding acquisition (equal), supervision (equal), writing – review and editing (equal).

## Funding

This work was funded by Hebei Agricultural University—Talent Introduction Project (YJ2025010 and YJ2025025); The Hebei Modern Agricultural Industry Technology System Flower Innovation Team (Chief Expert) Project of the Department of Agriculture and Rural Affairs of Hebei Province (HBCT2024200101); and The High‐end Talent Project of the Organization Department of the Hebei Provincial Party Committee (Yinran Huang).

## Ethics Statement

The authors have nothing to report.

## Consent

The authors have nothing to report.

## Conflicts of Interest

The authors declare no conflicts of interest.

## Supporting information


**Table S1:** SSR types in the six *Ulmus pumila* cultivars.

## Data Availability

The datasets generated and/or analyzed during the current study are available in the Mendeley Data repository (Wang 2026a, 2026b).
