# Supplementary material for: Complete Chloroplast Genomes of Five Ulmus pumila Cultivars: Comparative Analysis, Evolutionary Dynamics, and Germplasm Authentication Implications
Source: Ecol Evol. 2026 Sep 29;16(10):e74352. doi: 10.1002/ece3.74352 (PMC13623499; doi:10.1002/ece3.74352)
Supplement: Supplementary file 1 [file ECE3-16-e74352-s001.docx]

**Table S1. SSR types in the six *Ulmus pumila* cultivars.**

| **SSR type** | **Repeat unit** | **YG** | **YGNH-B** | **YGNH-G** | **YR** | **ZY** | **JM** |
| --- | --- | --- | --- | --- | --- | --- | --- |
| Mono- | A/T | 123 | 123 | 116 | 116 | 119 | 123 |
|  | C/G | 1 | 1 | 1 | 1 | 0 | 1 |
| Di- | AG/CT | 1 | 1 | 1 | 1 | 1 | 1 |
|  | AT/AT | 20 | 20 | 18 | 18 | 20 | 20 |
| Tri- | AAT/ATT | 8 | 8 | 7 | 7 | 8 | 8 |
|  | ACT/AGT | 1 | 1 | 1 | 1 | 1 | 1 |
| Tetra- | AAAT | 1 | 1 | 2 | 2 | 1 | 1 |
|  | AATA | 2 | 2 | 0 | 0 | 1 | 1 |
|  | TTTA | 1 | 1 | 3 | 3 | 1 | 2 |
|  | CTTT | 1 | 1 | 1 | 1 | 1 | 1 |
|  | TATT | 1 | 1 | 0 | 0 | 0 | 1 |
|  | ATAA | 1 | 1 | 0 | 0 | 1 | 1 |
|  | TTAT | 1 | 1 | 2 | 2 | 2 | 1 |
|  | TTTC | 0 | 0 | 0 | 0 | 1 | 1 |
| Penta- | TTATT | 1 | 1 | 0 | 0 | 1 | 1 |
|  | TAAAG | 1 | 1 | 1 | 1 | 1 | 1 |
| Hexa- | TGGTAG | 0 | 0 | 1 | 1 | 0 | 0 |
| **Total** |  | **164** | **164** | **154** | **154** | **159** | **164** |

Note: YGNH-B, *Ulmus pumila*‘Yangguang Nanhai’; YGNH-G,*Ulmus pumila*‘Yangguang Nühai’; XR, *Ulmus pumila*‘Xu Ri’; YG, *Ulmus pumila*‘Yang Gang’; ZY, *Ulmus pumila*‘Zhao Yang’;JM, *Ulmus pumila* Cultivar 'Jinmi'.
